# Supplementary material for: Identification of a fungal antibacterial endopeptidase that cleaves peptidoglycan
Source: EMBO Rep. 2025 Jul 4;26(15):3889–916. doi: 10.1038/s44319-025-00508-3 (PMC12332128; doi:10.1038/s44319-025-00508-3)
Supplement: Supplementary file 1 — Appendix [file 44319_2025_508_MOESM1_ESM.pdf]

Appendix

Identification of a fungal antibacterial endopeptidase that cleaves peptidoglycan

Silke Machata, Ute Bertsche, Franziska Hoffmann, Zaher M. Fattal, Franziska Kage, Michal Flak, Alexander N. J. Iliou, Falk Hillmann, Ferdinand von Eggeling, Hortense Slevogt, Axel A. Brakhage, Ilse D. Jacobsen

Table of Contents

**Appendix Table S1:**	Peptides predicted by *in silico* digestion and identified by MSI	**page 2**
**Appendix Table S2:**	Susceptibility of *S. aureus* strains to CwhA	**page 3**
**Appendix Table S3:**	Masses of peptides shown in Figure 4.	**page 4**
**Appendix Table S4**:	Multiresistant *S. aureus* (MRSA) strains used in this study.	**page 5**
**Appendix Table S5**:	Oligonuceotides used in this study	**page 6**
**Appendix Figure S1**	Muropeptide profiles	**page 7**

Appendix Table S1

m/z	Modification	Missed Cleavage	Peptide Sequence	MSI m/z valueswith adducts
1092.378	none	1	(K)ASRIPPLPLK(L)	1093.38558 [M+H]
1177.397	none	1	(R)TVFTPGIKASR(I)	1178.40468 [M+H]
2017.331	none	2	(R)HVPYSMNKVYPDPQGRK (Y)	2018.33858 [M+H]
1156.301	none	1	(K)KEYNTLECR(G)	1174.34443 [M+NH4]
1150.437	none	1	(-)MRTVFTPGIK(A)	1173.42652 [M+Na]
1061.277	1Met-loss+Acetyl	1	(-)MRTVFTPGIK(A)	1125.29277 [M+ACN+Na]

Appendix Table S1: Peptides predicted by *in silico* digestion and identified by MALDI-imaging mass spectrometry (MSI) in both lung sections and control spots containing recombinant CwhA. Distribution of these peptides closely matched the example of peptide (K)KEYNTLECR(G) shown in Figure 1.

Appendix Table S2

Strain	PBS	CwhA	*P* value
ATCC13420	92.9 ± 1.7	74.7 ± 1.1	0.0078
MRSA 124 722	92.0 ± 0.0	68.0 ± 1.7	0.0017
MRSA 124 622	93.7 ± 5.8	62.0 ± 1.7	0.0054
MRSA124 737	93.7 ± 0.0	70.0 ± 3.5	0.0128
MRSA 293 888	93.0 ± 1.7	68.7 ± 4.0	0.0075
MRSA 94 003	96.0 ± 1.7	78.7 ± 1.1	0.0004
MRSA 314 432	91.0 ± 2.6	77.7 ± 4.7	0.0857
MRSA 124 940	90.7 ± 7.5	71.3 ± 9.2	0.1835
MRSA 124 411	92.0 ± 1.7	72.7 ± 11.5	0.1278
MRSA 124 328	92.7 ± 4.0	69.7 ± 11.0	0.0289

Appendix Table S2: Susceptibility of *S. aureus* strains to CwhA. Relative optical density (600 nm, normalized to 0 min) of *S. aureus* strains after 120 min incubation in PBS or 100 mg/ml CwhA. Mean ± SD of values obtained in three independent experiments; p value: Comparison of PBS and CwhA treatment by two-tailed paired t-test. *P* values < 0.05 are highlighted by a light grey background.

Appendix Table S3

Peak	Measured $[M+H]^+$	Basic structure	Variations			
			GlcNac missing	additional O-Acetylation	amidated mDpm	N-Acetylation missing
		S. aureus				
1	968.4781	DS-Pentapeptide				
2	1253.5867	1xDS-Pentapeptide-Gly5				
3	2417.1157	2xDS-Pentapeptide-Gly5				
4	3580.6537	3xDS-Pentapeptide-Gly5				
5	2328.0688	Cyclic 2xDS-Tetrapeptide-Gly5				
P	2027.0106	Peptide part of a tetramer				
P	2511.2478	Peptide part of a tetramer				
P	2995.4873	Peptide part of a tetramer				
P	3479.72915	Peptide part of a tetramer				
Dx	495.2292	1xDS-Dipeptide	1			
D1	698.3086	1xDS-Dipeptide				
Dy	740.3195	1xDS-Dipeptide		1		
D2	1375.58432	2xDS-Dipeptide				
D3	2052.85794	3xDS-Dipeptide				
D4	2730.13284	4xDS-Dipeptide				
D5	3407.41374	5xDS-Dipeptide				
D6	4084.68696	6xDS-Dipeptide				
D7	4761.97016	7xDS-Dipeptide				
D8	5439.25176	8xDS-Dipeptide				
D9	6116.53188	9xDS-Dipeptide				
		B. subtilis				
1	870.3922	DS-Tripeptide			1	
2	871.3762	DS-Tripeptide				
3	1750.7930	DS-Tetrapeptide - DS-Tripeptide			2	1
4	1751.7770	DS-Tetrapeptide - DS-Tripeptide			1	1
5	1792.8042	DS-Tetrapeptide - DS-Tripeptide			2	
6	1752.7612	DS-Tetrapeptide - DS-Tripeptide				1
7	1793.7890	DS-Tetrapeptide - DS-Tripeptide			1	
8	1793.7878	DS-Tetrapeptide - DS-Tripeptide			1	
9	1794.7544	DS-Tetrapeptide - DS-Tripeptide				
Dz	657.2823	DS-Dipeptide				1
D1	699.2931	DS-Dipeptide				

Appendix Table S3: Masses of peptides shown in Figure 4.

Appendix Table S4

Strain no.	Strain type	Origin
124 722	CC5/ST228-MRSA-I, Süddeutscher	human
124 622	CC133-MSSA (lukF-P83/lukM+)	Veterinär
124 737	CC8-MRSA-IVh/j (sea+), "UK-EMRSA-2"	human
293 888	CC398-MRSA-[V/VT+ccrB1]	veterinary
94 003	CC5-MRSA-II (tst1+), New York-Japan Clone	Mu50 / reference strain
314 432	CC772-MRSA-V/VT (PVL+)	human
124 940	CC80-MRSA-IVc (PVL+)	human
124 411	CC8-MRSA-[IV+ACME] (PVL+), USA300	human
124 328	CC22-MRSA-IV, UK-EMRSA-15/Barnim EMRSA	human

Appendix Table S4: Multiresistant *S. aureus* (MRSA) strains used in this study. Strains are part of the strain collection of the University Hospital Dresden, Germany, and were kindly provided by S. Monecke.

Appendix Table S5

Name	Sequence 5' ⟶ 3'
Del_p60A_F1	CACGACGTTGTAAAACGACGGCCAGTGCCAGGTATAGCGAGGTTGCATCC
Del_p60A_R2	GAGGCCATCTAGGCCATCAAGCGCGAGCAGAGTTGACAAGTG
Del_p60A_F3	GGCCTGAGTGGCCATCGAATTCGATGGAGCGTTGGATAGGTG
Del_p60A_R4	GATCCTCTAGAGTCGACCTGCAGGCATGCAGAAGCCATTCTCTGCTATTC
ptrA_for_II	GAATTCGATGGCCACTCAGGCC
ptrA_rev_II	GCTTGATGGCCTAGATGGCCTC
o_p60AFor	GAGGATCCGTACCCCATCACTGGCAACG
o_p60ARev	GAGAATTCTTAGTCCACAACGCG
Expr_p60A_for	ATGCGCACTGTATTCACTCC
Expr_p60A_rev	CATGATATCTTAGTCCACAACGCGGATGTA
RT_p60Afor	TGGCTTCAGCACTGTCACTC
RT_p60Arev	TGTACTTGACGCAGCCGTAG
RT_Cox5 for	ATCTGTTCGCCAAGCCCAAG
RT_Cox5 rev	TCACTGCTGACACCGTAGAG
RT_act1_for	CCACGTCACCACTTTCAACTC
RT_act1_rev	CTGCATACGGTCGGAGATAC

Appendix Table S5: Oligonuceotides used in this study.

Appendix Figure S1

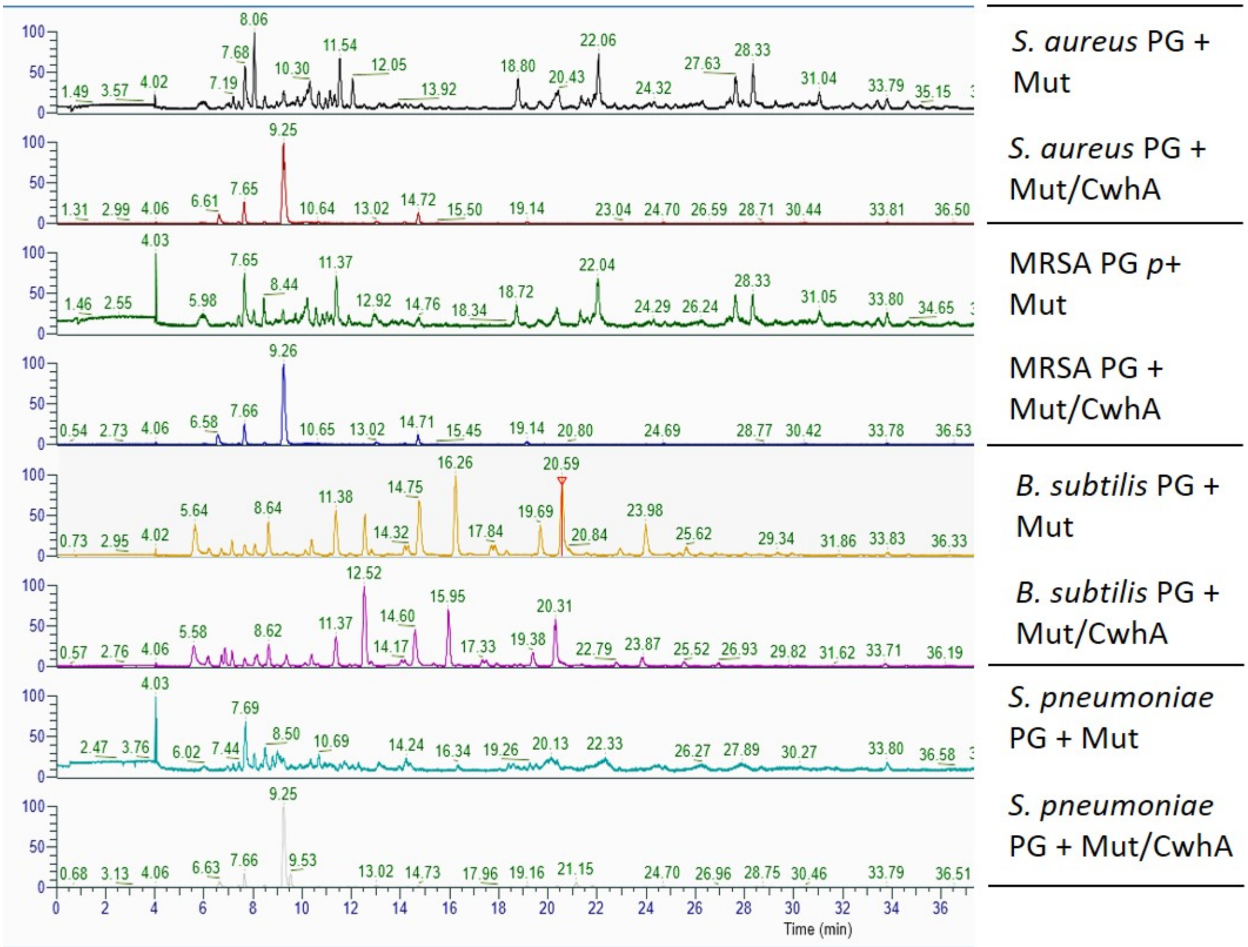

Appendix Figure S1. Muropeptide profile of peptidoglycan from *S. aureus* SA113, MRSA 181*, S. pneumoniae* (Klein 1884), *B. subtilis* (Ehrenberg 1835) obtained by UPLC/MS after treatment with mutanolysin or/and CwhA. Masses of peaks are shown in Supplementary Table 3. The asterix indicates peaks that are variants of Tetra-Tri.
