# Supplementary figures and images for: Identification of a fungal antibacterial endopeptidase that cleaves peptidoglycan
Source: EMBO Rep. 2025 Jul 4;26(15):3889–916. doi: 10.1038/s44319-025-00508-3 (PMC12332128; doi:10.1038/s44319-025-00508-3)

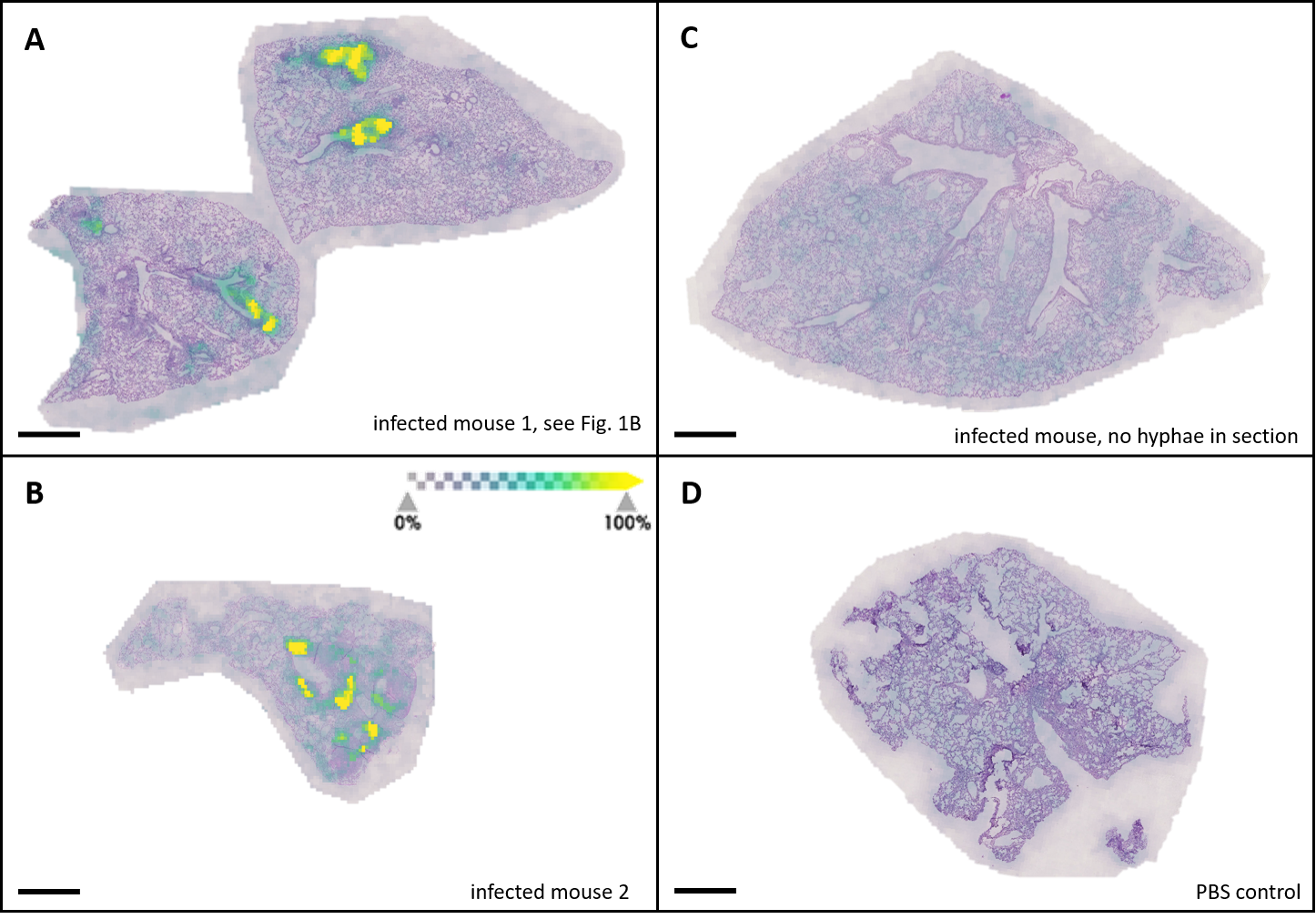

Supplement: Supplementary file 5 — Source data Fig. 1 [file 44319_2025_508_MOESM5_ESM.zip › EMBOR-2024-60365V2__SourceDataForFigure 1B.tif]

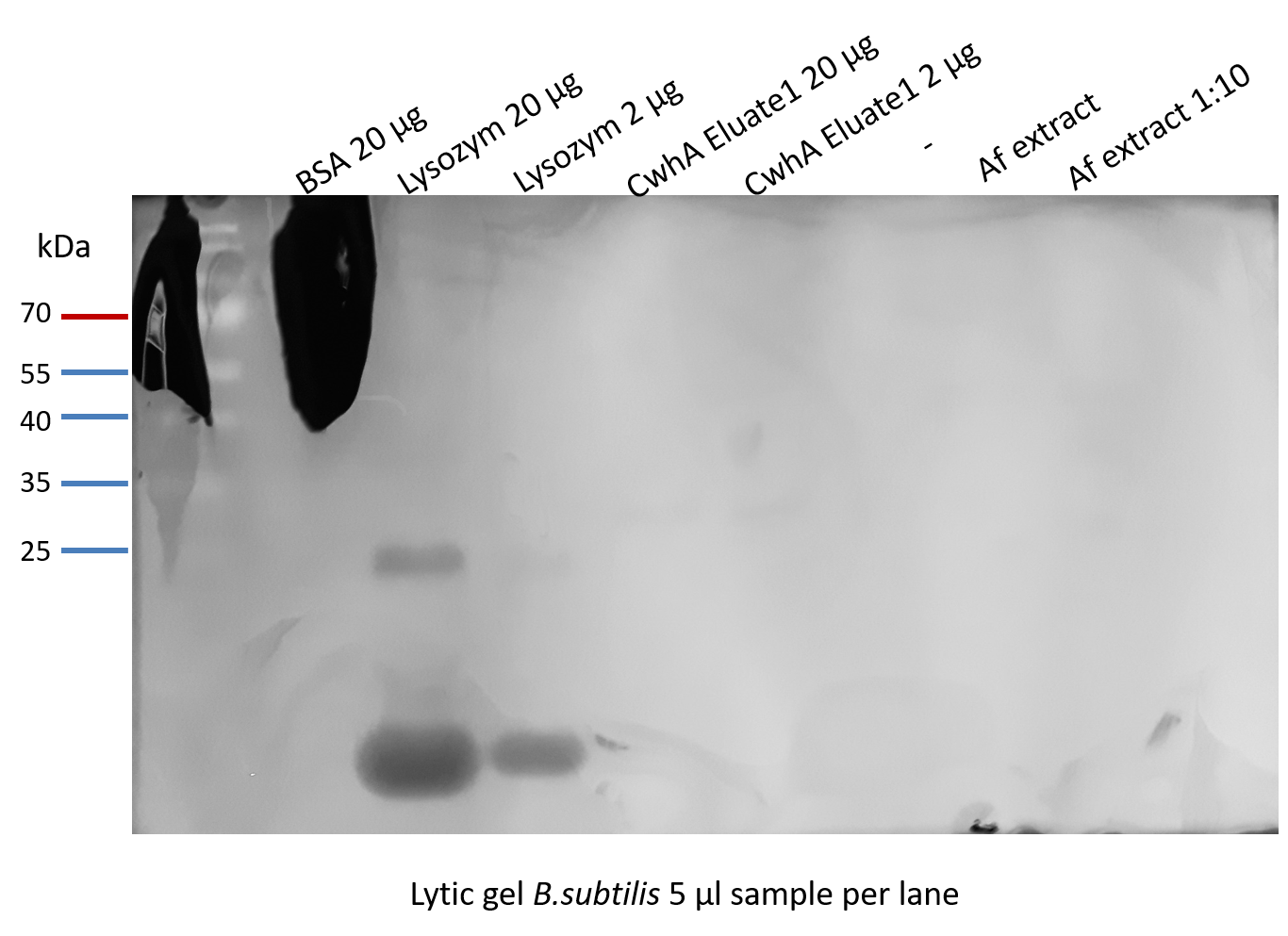

Supplement: Supplementary file 6 — Source data Fig. 2 [file 44319_2025_508_MOESM6_ESM.zip › EMBOR-2024-60365V2__SourceDataForFigure 2B/B subtilis_lytic gel_170816.tif]

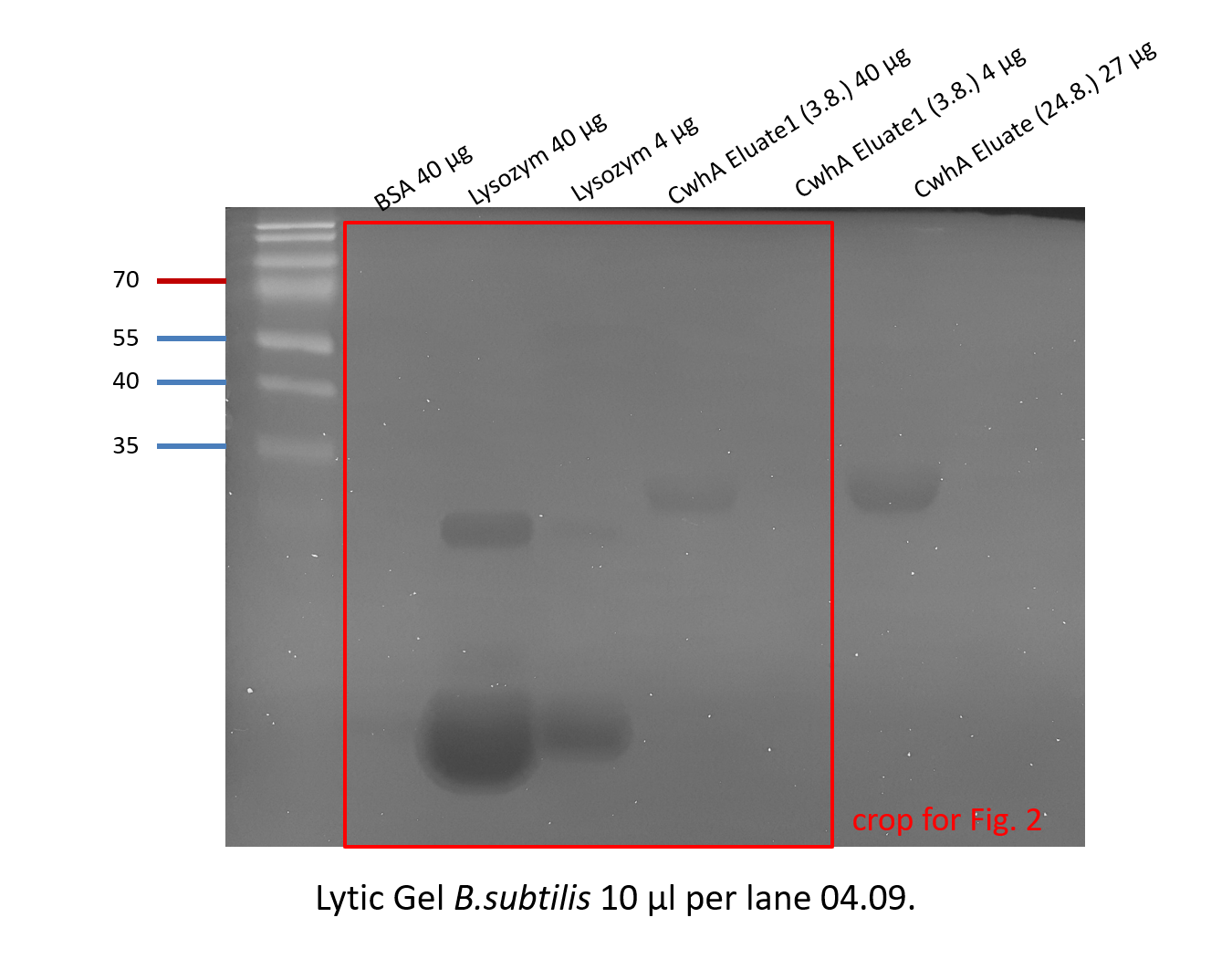

Supplement: Supplementary file 6 — Source data Fig. 2 [file 44319_2025_508_MOESM6_ESM.zip › EMBOR-2024-60365V2__SourceDataForFigure 2B/B subtilis_lytic gel_170904.tif]

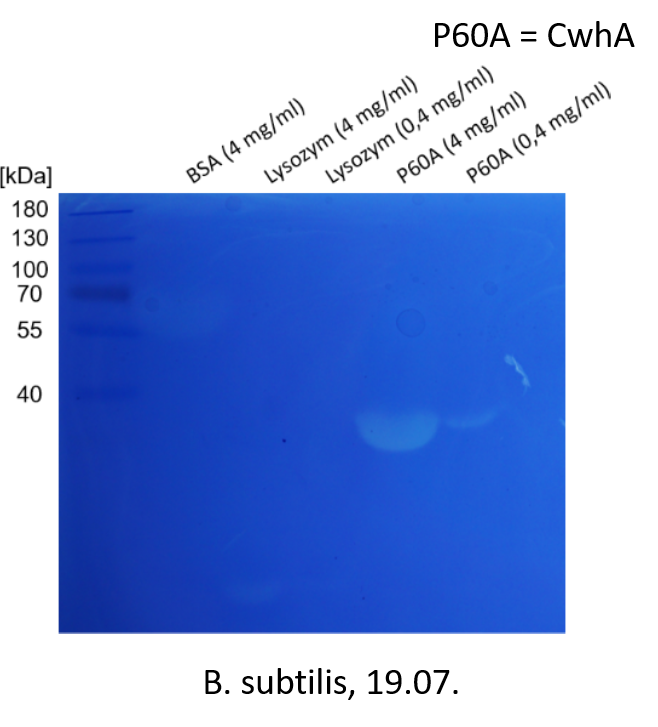

Supplement: Supplementary file 6 — Source data Fig. 2 [file 44319_2025_508_MOESM6_ESM.zip › EMBOR-2024-60365V2__SourceDataForFigure 2B/B subtilis_lytic gel_180719.tif]

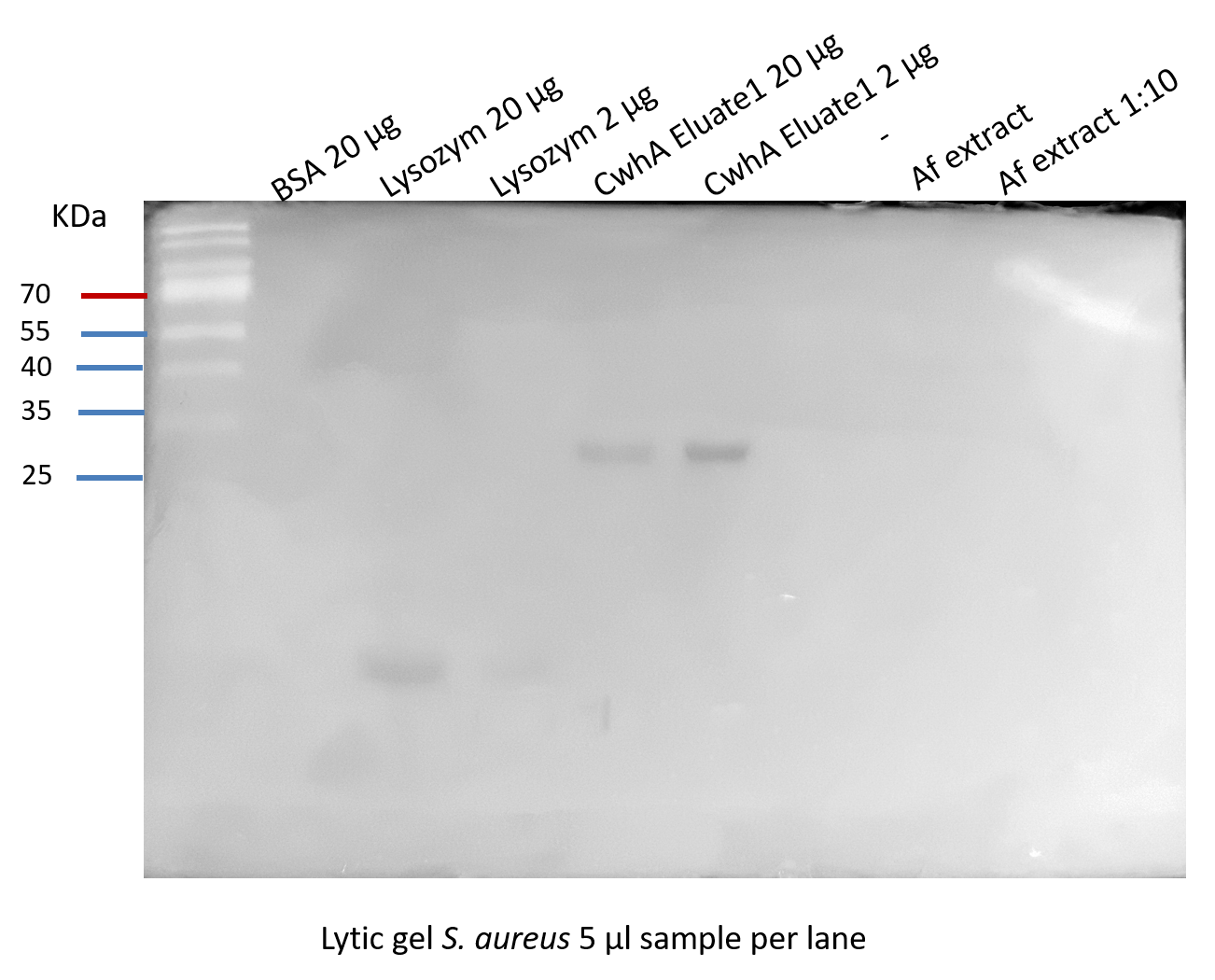

Supplement: Supplementary file 6 — Source data Fig. 2 [file 44319_2025_508_MOESM6_ESM.zip › EMBOR-2024-60365V2__SourceDataForFigure 2B/S aureus_lytic gel_170816.tif]

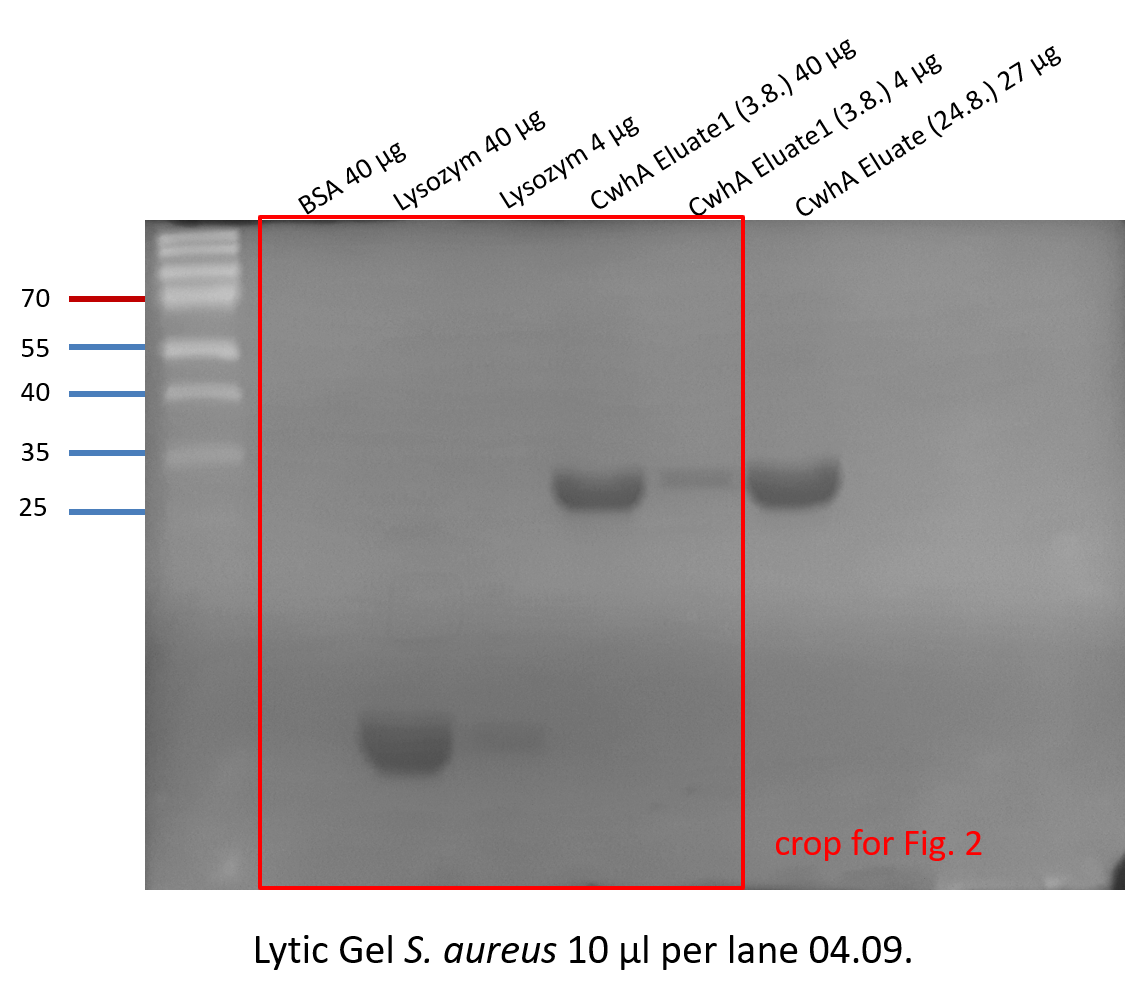

Supplement: Supplementary file 6 — Source data Fig. 2 [file 44319_2025_508_MOESM6_ESM.zip › EMBOR-2024-60365V2__SourceDataForFigure 2B/S aureus_lytic gel_170904.tif]

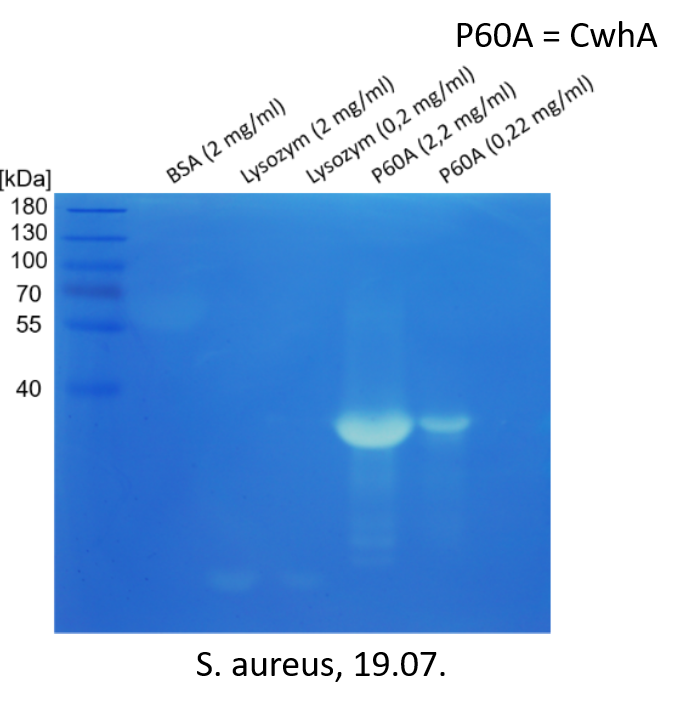

Supplement: Supplementary file 6 — Source data Fig. 2 [file 44319_2025_508_MOESM6_ESM.zip › EMBOR-2024-60365V2__SourceDataForFigure 2B/S aureus_lytic gel_180719.tif]

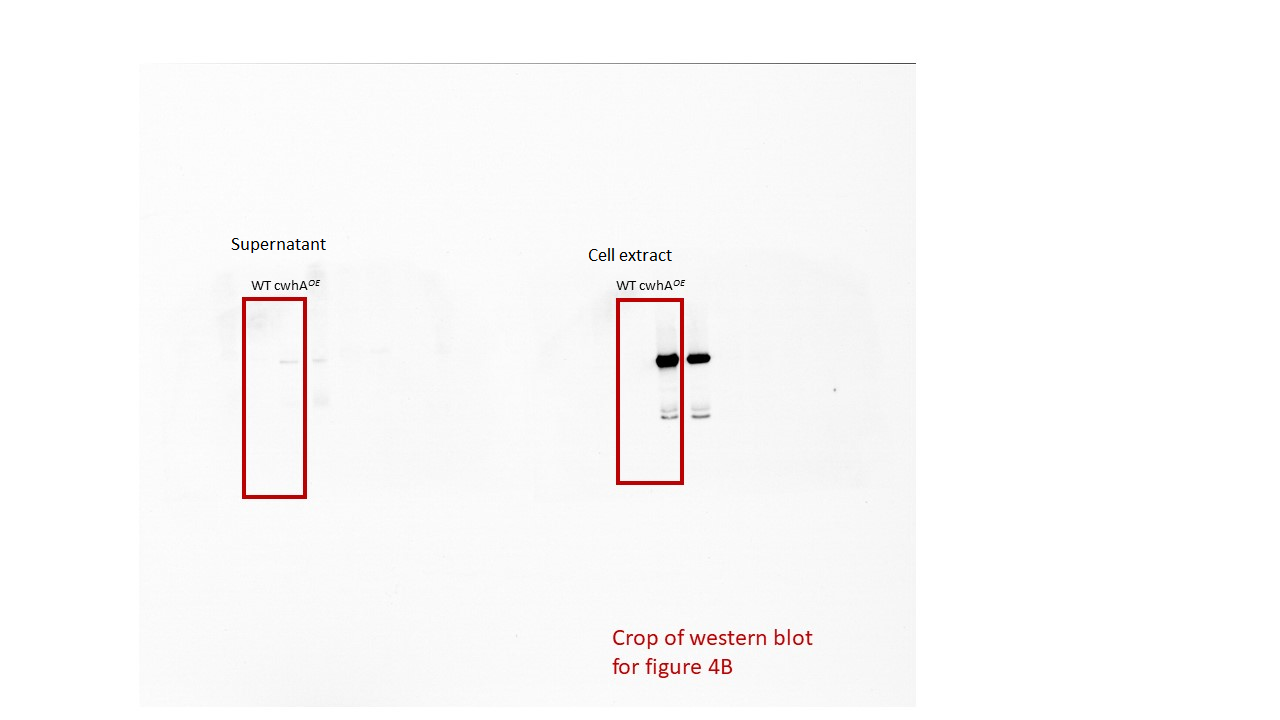

Supplement: Supplementary file 8 — Source data Fig. 4 [file 44319_2025_508_MOESM8_ESM.zip › EMBOR-2024-60365V3__SourceDataForFigure 4/Crop4B.tif]

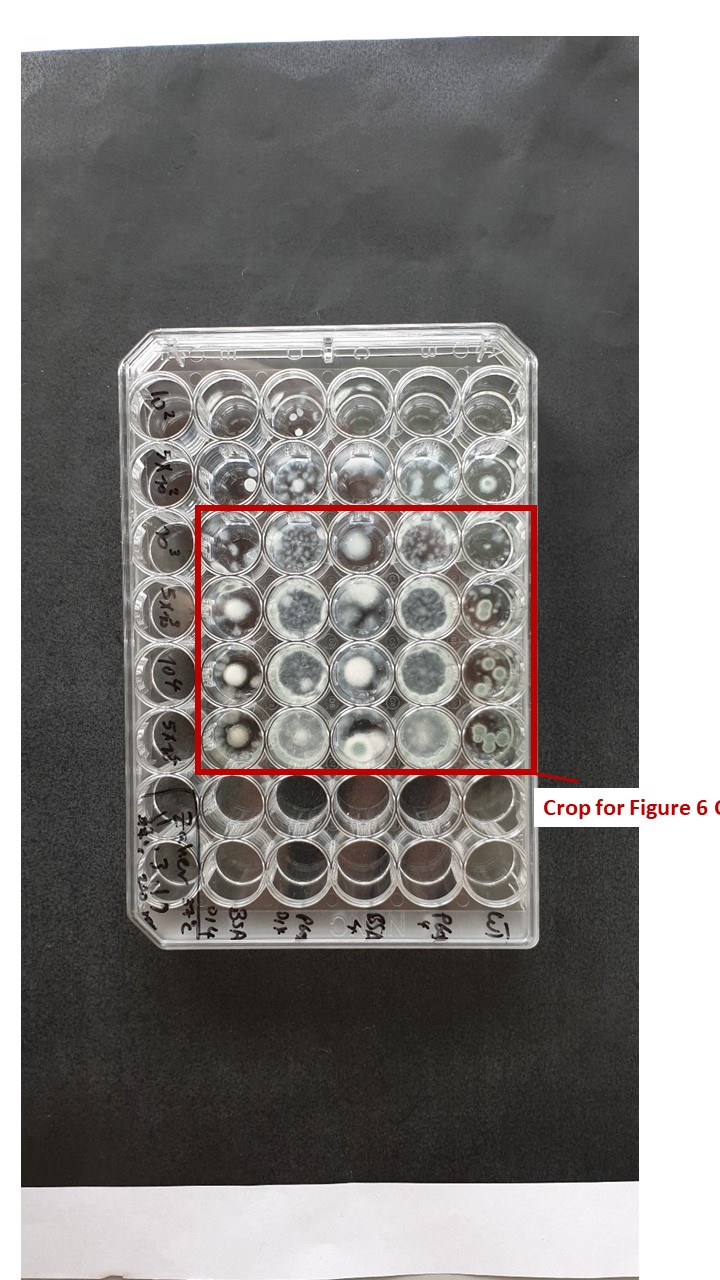

Supplement: Supplementary file 10 — Source data Fig. 6 [file 44319_2025_508_MOESM10_ESM.zip › EMBOR-2024-60365V3__SourceDataForFigure 6/EMBOR-2024-60365V2__SourceDataForFigure6C.tif]

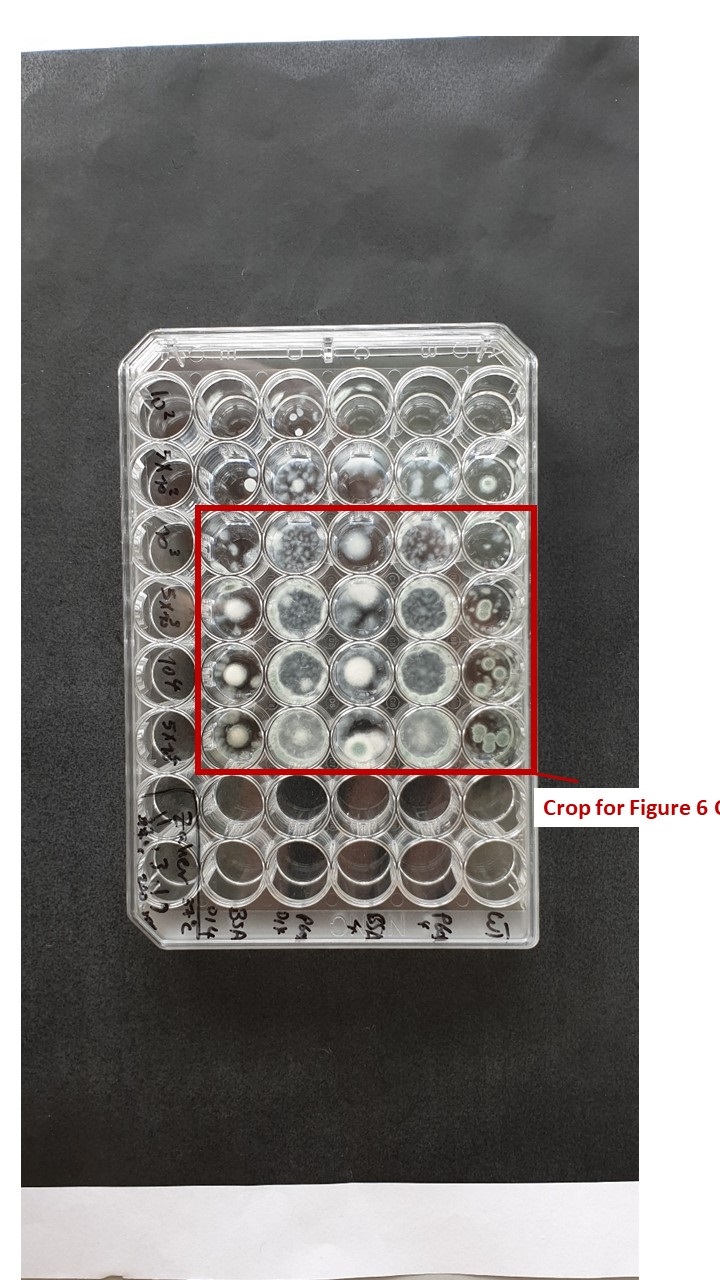

Supplement: Supplementary file 10 — Source data Fig. 6 [file 44319_2025_508_MOESM10_ESM.zip › EMBOR-2024-60365V3__SourceDataForFigure 6/Figure6C_original_CROP.jpg]
