# Supplementary material for: Identification of a fungal antibacterial endopeptidase that cleaves peptidoglycan
Source: EMBO Rep. 2025 Jul 4;26(15):3889–916. doi: 10.1038/s44319-025-00508-3 (PMC12332128; doi:10.1038/s44319-025-00508-3)
Supplement: Supplementary file 13 — Expanded View Figures [file 44319_2025_508_MOESM13_ESM.pdf]

Expanded View Figures

Figure EV1. Effects of CwhA on bacterial growth in LB media and Tris buffer.

(**A**) Bacterial growth in LB media supplemented with CwhA. The optical density at 600 nm was measured every 30 min in a plate reader after the addition of PBS or different concentrations of recombinant CwhA (25–200 µg/ml). One representative experiment is shown. (**B**) The optical density (600 nm) after addition of recombinant CwhA (16–200 µg/ml) to bacterial suspensions in 50 mM Tris buffer (pH 8) was measured overtime. Data from a single experiment is shown. Data from a biological replicate is shown in Fig. 3A. Source data are available online for this figure.

© The Author(s)

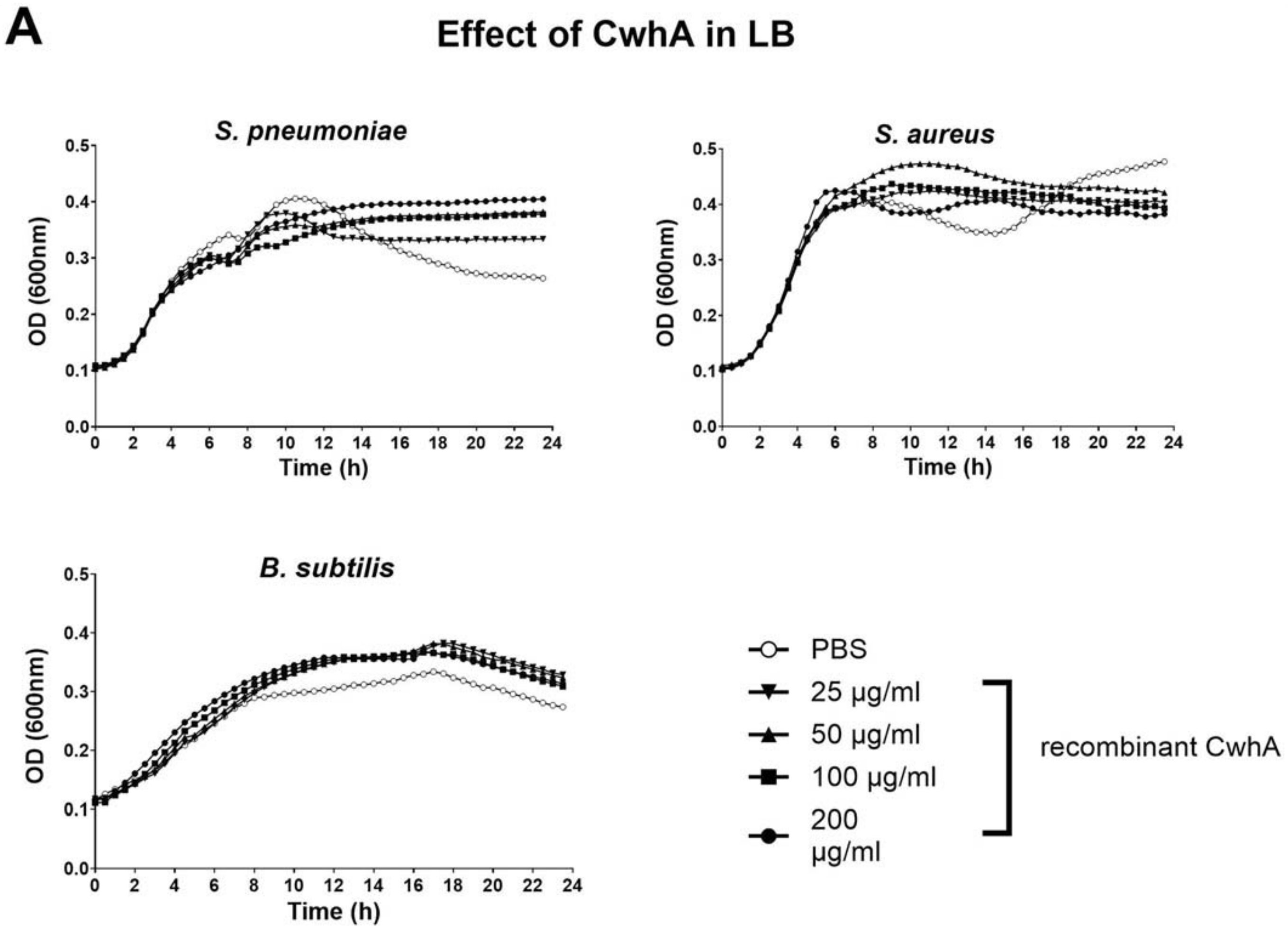
A
Effect of CwhA in LB
S. pneumoniae
S. aureus
B. subtilis
OD (600nm)
Time (h)
PBS
25 µg/ml
50 µg/ml
100 µg/ml
200 µg/ml
recombinant CwhA

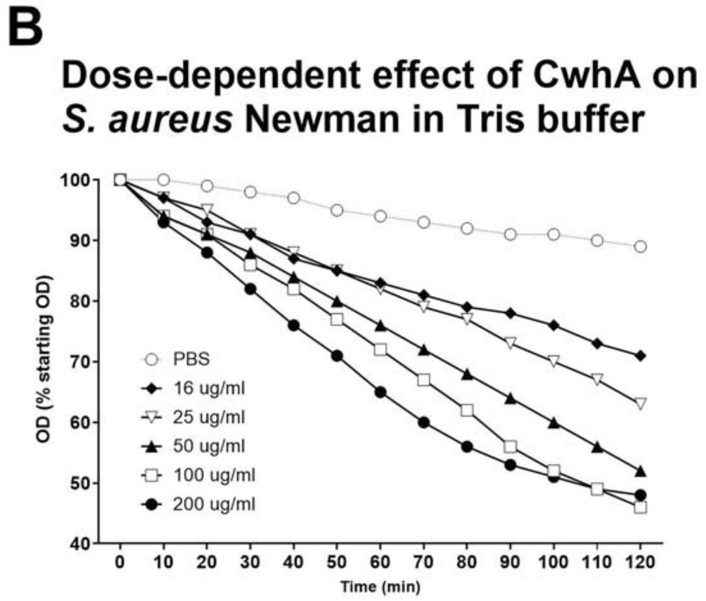
B
Dose-dependent effect of CwhA on S. aureus Newman in Tris buffer
OD (% starting OD)
Time (min)
PBS
16 ug/ml
25 ug/ml
50 ug/ml
100 ug/ml
200 ug/ml

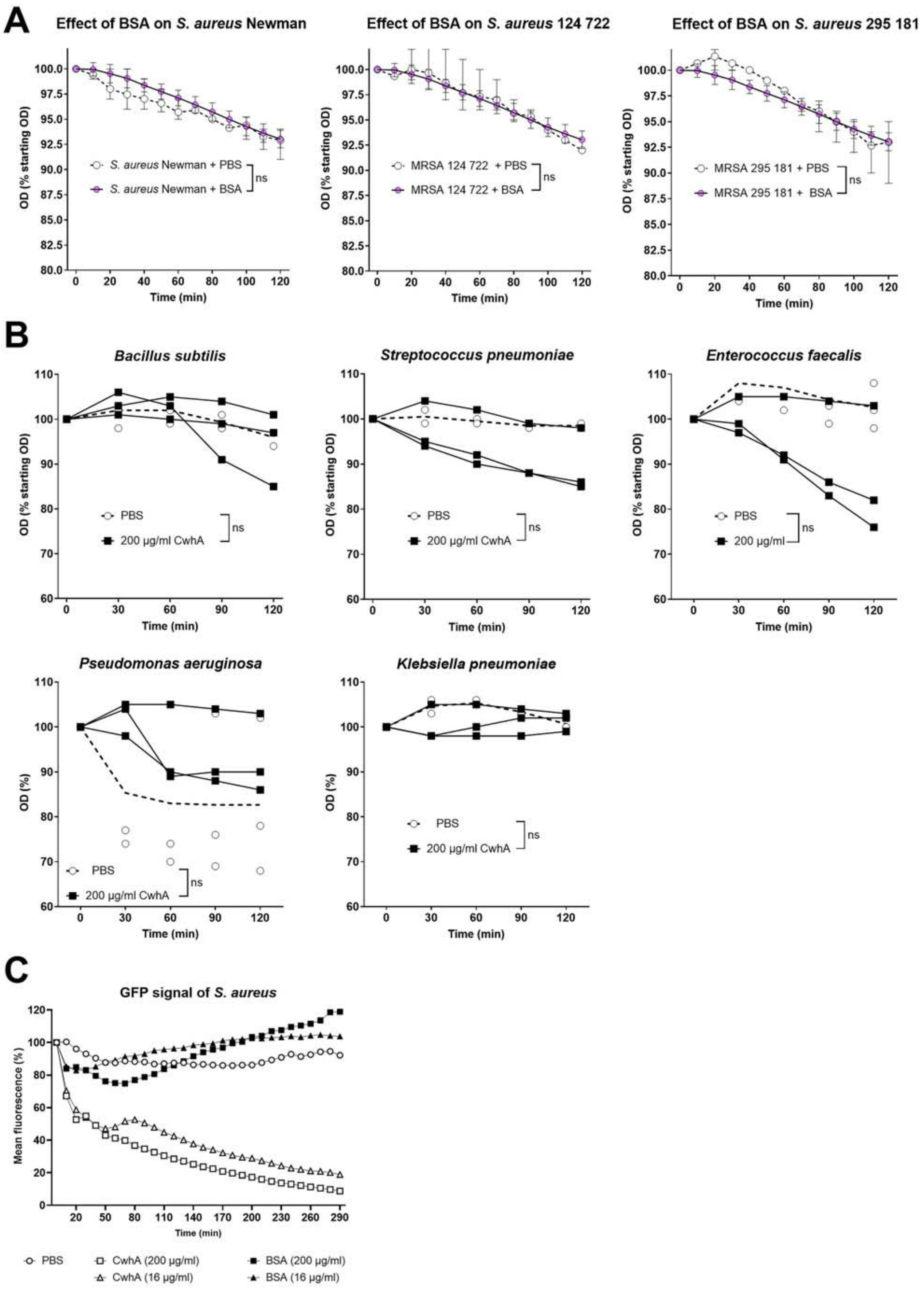
A
Effect of BSA on S. aureus Newman
S. aureus Newman + PBS
S. aureus Newman + BSA
ns
Effect of BSA on S. aureus 124 722
MRSA 124 722 + PBS
MRSA 124 722 + BSA
ns
Effect of BSA on S. aureus 295 181
MRSA 295 181 + PBS
MRSA 295 181 + BSA
ns
OD (% starting OD)
Time (min)
B
Bacillus subtilis
Streptococcus pneumoniae
Enterococcus faecalis
PBS
200 µg/ml CwhA
200 µg/ml
ns
Pseudomonas aeruginosa
Klebsiella pneumoniae
OD (%)
C
GFP signal of S. aureus
Mean fluorescence (%)
Time (min)
PBS
CwhA (200 µg/ml)
CwhA (16 µg/ml)
BSA (200 µg/ml)
BSA (16 µg/ml)

 © The Author(s)

Figure EV2. Effect of BSA on *S. aureus* and CwhA-mediated lysis of different bacterial species.

(**A**, **B**) The optical density (OD) at 600 nm after addition of PBS, BSA (200 mg/ml) or recombinant CwhA (200 mg/ml) to bacterial suspensions ($n = 3$ biological replicates) in 50 mM Tris buffer (pH 8) was measured at the indicated time points. (**A**) Three different *S. aureus* strains in Tris buffer after addition of either PBS or BSA. The PBS data is also shown in Fig. 3B. (**B**) OD of different bacterial species after the addition of either PBS or CwhA. (**C**) GFP signal of a GFP-expressing *S. aureus* strain after treatment with CwhA. Bovine serum albumin (BSA) and PBS were used as controls. One of $n = 2$ biological replicates, see also Fig. 3D. Data information: In (**A**, **B**) data were shown as individual data points and the mean as connecting line. ns no statistically significant difference between the groups (two-way ANOVA). Source data are available online for this figure.

© The Author(s)

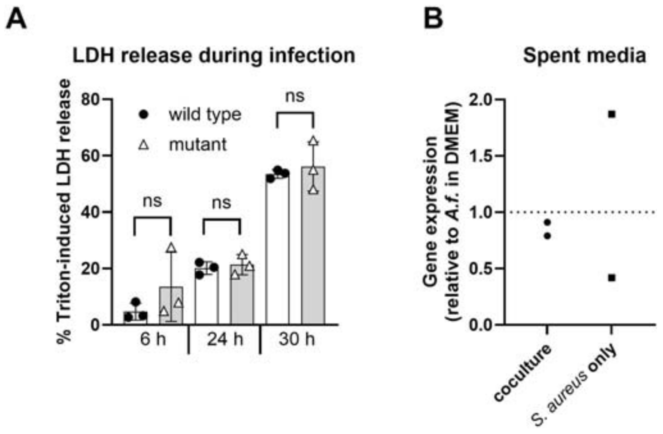

Figure EV3. Host cell damage during *A. fumigatus* infection, and the effect of spent media on *cwhA* expression.

(**A**) A549 cells were seeded in 24-well microplates and infected with 9×10^5 *A. fumigatus* conidia. Plates were incubated at 37 °C, 5% CO_2 and 21% O_2 for either 6, 24, or 30 h. LDH released into the supernatant of infected cells was normalized to the Triton-lysed positive control. $n = 3$ biological replicates per treatment and time point. (**B**) *A. fumigatus* was grown in spent media (sterile-filtered culture supernatant) of *S. aureus* Newman cultured alone or in coculture with *A. fumigatus* for 4 h in DMEM. $n = 2$ biological replicates. Data information: Data in (**A**) are shown as a scatter plot with bars (mean) and SD (error bars). ns not significant (two-tailed unpaired *t*-test). In (**B**), data were represented as individual data points. Source data are available online for this figure.

 © The Author(s)

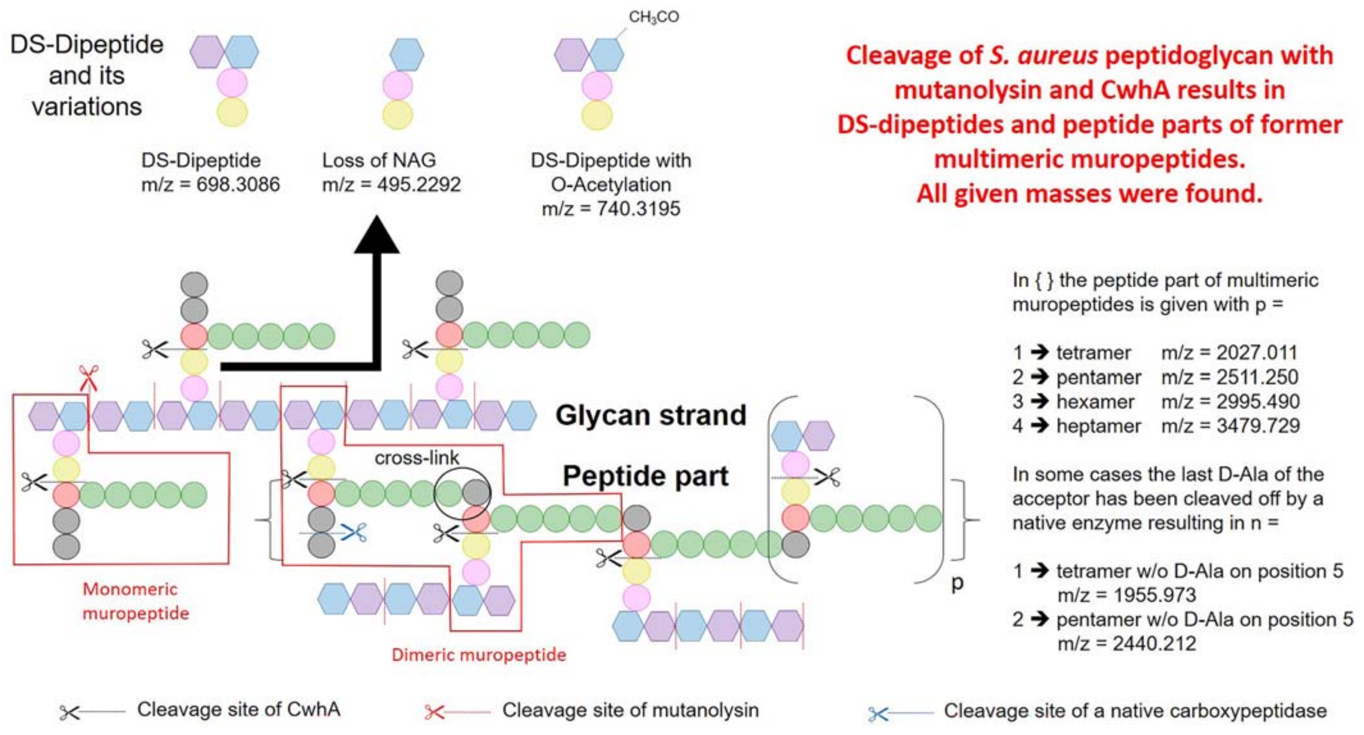

Figure EV4. Schematic depiction of peptidoglycan cleavage by mutanolysin and CwhA.

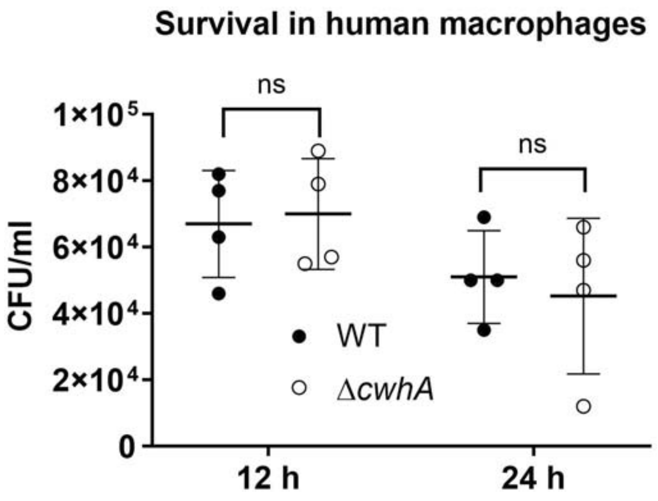

Figure EV5. Survival of *Aspergillus fumigatus* wildtype (WT) and Δ*cwhA* in human macrophages.

Differentiated adherent human monocyte-derived macrophages ($n = 4$ different donors - biological replicates) were infected with fungal conidia at an MOI of 1 for 12 and 24 h and lysed to determine fungal growth on malt agar plates. Data information: Data from four independent donors are shown as a scatter dot plot with mean ± SD. ns not significant (one-way ANOVA and Tukey's multiple comparisons). Source data are available online for this figure.

© The Author(s)
